# Machine learning in point-of-care automated classification of oral potentially malignant and malignant disorders: a systematic review and meta-analysis

**DOI:** 10.1038/s41598-022-17489-1

**Published:** 2022-08-13

**Authors:** Ashley Ferro, Sanjeev Kotecha, Kathleen Fan

**Affiliations:** 1 Faculty of Dentistry, Oral and Craniofacial Sciences, King’s College London, London, UK; 2 Oral and Maxillofacial Surgery Department, King’s College Hospital NHS Foundation Trust, Denmark Hill, London, SE1 9RT UK

**Keywords:** Cancer, Cancer prevention, Cancer screening, Computational science

## Abstract

Machine learning (ML) algorithms are becoming increasingly pervasive in the domains of medical diagnostics and prognostication, afforded by complex deep learning architectures that overcome the limitations of manual feature extraction. In this systematic review and meta-analysis, we provide an update on current progress of ML algorithms in point-of-care (POC) automated diagnostic classification systems for lesions of the oral cavity. Studies reporting performance metrics on ML algorithms used in automatic classification of oral regions of interest were identified and screened by 2 independent reviewers from 4 databases. Preferred Reporting Items for Systematic Reviews and Meta-Analyses (PRISMA) guidelines were followed. 35 studies were suitable for qualitative synthesis, and 31 for quantitative analysis. Outcomes were assessed using a bivariate random-effects model following an assessment of bias and heterogeneity. 4 distinct methodologies were identified for POC diagnosis: (1) clinical photography; (2) optical imaging; (3) thermal imaging; (4) analysis of volatile organic compounds. Estimated AUROC across all studies was 0.935, and no difference in performance was identified between methodologies. We discuss the various classical and modern approaches to ML employed within identified studies, and highlight issues that will need to be addressed for implementation of automated classification systems in screening and early detection.

## Introduction

Head and neck cancer (HNC), including of the oral cavity, oropharynx, hypopharynx and larynx, is currently the sixth most-common malignancy worldwide, with over 60,000 cases in 2020 in the United States alone^1^. Squamous cell carcinoma accounts for over 90% of cases of cancer of the oral cavity and, despite increasing awareness of modifiable risk factors, its incidence continues to increase^2^. Standard treatment for localised cancers of the oral cavity is surgical resection, oftentimes accompanied by neck dissection and flap reconstruction. Although offering a prospect of disease resolution, these radical resections are associated with significant morbidity, including swallowing and articulation difficulties, reduced mobility, chronic pain, significant disfigurement, and the accompanying psychosocial impact inherent to these complications^3^.

Oral Squamous Cell Carcinoma (OSCC) develops through a series of well-established molecular events secondary to the interplay between genetic predisposition and exposure to environmental carcinogens. The progressive acquisition of mutations in proto-oncogenes and tumour suppressor genes with continued carcinogen exposure is reflected through a sequence of dysplasia to neoplasia, and accompanied by gross morphological changes in the oral mucosa^4^. Unfortunately, many potentially malignant disorders and early malignancies are asymptomatic and subtle, resulting in late presentation and suboptimal outcomes^5^.

Definitive gold-standard diagnosis of oral potentially malignant and malignant disorders is dependent upon biopsy and histopathological evaluation of haematoxylin and eosin-stained sections. This is both invasive and time-intensive, requiring the expertise of consultant histopathologists for accurate diagnosis. Limited access to expensive laboratory resources and histopathology expertise is a particular concern for low and middle-income countries, areas disproportionally afflicted by OSCC^6,7^. There is thus a clear need for the development of non-invasive point-of-care (POC) screening tools for early HNC detection that do not so heavily rely on expertise for sample preparation and interpretation. Machine learning may provide the solution to this conundrum.

Machine learning, as a domain of artificial intelligence, involves the ability of an algorithm to *learn* information and draw inferences from patterns within data without explicit programmed instruction (Supplemental Table S1). Driven by advancements in computational power and algorithm efficiency, the last decade has witnessed a rapid increase in the complexity of these algorithms. The emergence of artificial neural networks, architectures mirrored on the structure of the human brain, paved the way for deep learning, a subfield of machine learning characterised by multi-layered neural networks capable of automatic feature extraction. These systems have already demonstrated exceptional performance in a range of different classification tasks in oncology, including prediction of diagnosis, prognosis and treatment response in a range of different malignancies^8^. In the current review, we summarise the current progress of machine learning in POC detection methods for potentially malignant and malignant disorders of the oral cavity, with a particular focus on methods of classification.

## Material and methods

This study was completed in keeping with the Preferred Reporting Items for Systematic Reviews and Meta-Analysis (PRISMA) guidelines.

### Search strategy

A systematic literature search was performed on 13 February 2022 using the following databases: PubMed, Embase, the Cochrane Central Register of Controlled Trials, and DBLP (computer science bibliography). The following terms were combined to identify relevant records: “artificial intelligence”, “machine learning”, “deep learning”, “neural network”, “artificial neural network”, “convolutional neural network”, “generative adversarial network”, “transfer learning”, “oral cancer”, “oral malignancy”. Additional records were retrieved by iteratively scrutinising reference lists of relevant publications.

### Inclusion criteria

Publications were selected for review if they satisfied the following inclusion criteria: full texts available in English language; studies using machine learning (of any class) to provide POC diagnostic information on intra-oral lesions of interest; studies providing outcomes of model performance compared to a human-determined ground truth (gold standard). Ground truth was considered ‘human-determined’ where annotations (upon which algorithms were trained and tested against) were made solely based on human histopathologist interpretation of tissue biopsies or through human interpretation of captured images where biopsies were not indicated.

### Exclusion criteria

The following exclusion criteria were applied: studies where human ground truth was not explicitly confirmed; studies providing only prognostic data; studies providing outcome data on mixed malignancies, where outcomes could not be extracted independently for oral pathology; studies incorporating clinical/demographic data into predictive models (models not based solely on the detection method), studies where the ML class was not explicitly stated; review articles, commentaries and expert opinions, and animal studies. Articles relating to machine learning based on radiological imaging (magnetic resonance imaging, computed tomography, positron emission tomography) and biomarkers were excluded, including those studies where additional manual sample processing is required before automatic classification (exfoliative cytology and brush biopsies).

### Data collection

Titles, abstracts and full texts were independently assessed by two reviewers. Discrepancies were resolved by consensus following discussion between reviewers to minimise selection bias. A custom data collection form was used to extract the following data: study title; authors; year of publication, title, category of test, sample source, sample size of control, sample size of suspicious lesion/region of interest, ground truth, lesion location, AI class, and performance metric. Sample size of the test set, for the purposes of downstream analysis, was assumed as the total number of analysed whole images of a given class (ROI vs control). Where a study presents multiple models, outcomes from the best-performing model were extracted for downstream analysis.

### Assessment of risk of bias

Assessment of bias from identified studies was determined using the QUADAS-2 tool, a scoring system developed for assessing risk of bias in studies of diagnostic accuracy^9^. Four domains are assessed through this scoring system: patient selection; index test; reference standard; and flow and timing. Risk of bias is judged as ‘low’, ‘high’ or ‘unclear’ according to scoring in these domains. Discrepancies in scoring between reviewers were resolved through consensus. No studies were excluded on the ground of risk of bias; instead, risk of bias was highlighted. Deek’s funnel plots were used to assess for publication bias across all studies and within each subgroup, and Egger’s regression test was used as a quantitative method to test for funnel plot asymmetry. The Duval and Tweedie trim and fill method was used to further examine small-study effects and estimate the magnitude of small study bias^10^. Rücker’s Limit meta-analysis method was additionally used to test for small-study effects, for both the main analysis (with all studies) and within each subgroup.

### Statistical analysis

Heterogeneity of outcomes between studies was assessed using Tau^2^, and Higgin’s *I*^2^ was used to assess the proportion of true variance of a weighted outcome. *I*^2^ was interpreted according to the Cochrane Collaboration, where 0–40% was considered as low heterogeneity, 30–60% as moderate heterogeneity, 50–90% as substantial heterogeneity and > 75% as considerable heterogeneity^11^. A Cochrane Q statistic p-value < 0.10 was accepted as significant. Forest plots for sensitivity and specificity were also used as a visual proxy of heterogeneity, following a univariate random-effects meta-analysis using a logit transformation. Since pooling of sensitivities and specificities across studies may be misleading, univariate approaches to meta-analyses of diagnostic test performance are not recommended. Thus, a bivariate random-effects model for logit-transformed pairs of sensitivities and false positive rates was used to provide an estimate of diagnostic test performance^12^. Performance is given as AUROC, and presented as summary ROC (sROC) curves with 95% confidence regions for the optimum performance threshold. Performance between different testing modalities, lesion type (e.g. OSCC vs benign), and AI type was visually assessed by comparing sROC curves and their respective confidence domains, before subgroup analysis through a bivariate diagnostic meta-regression.

Patterns of heterogeneity were further explored through the use of Graphic Display of Study Heterogeneity (GOSH) plots for both sensitivity and specificity independently, using a maximum of 1 × 10^6^ randomly fitted models given computational demand^13^. Influential outlying studies were then inferred through unsupervised clustering (*k-*means clustering, density-based spatial clustering of applications with noise (DBSCAN), and Gaussian mixture models) of GOSH plot data. Cooke’s distance was used to determine the influence of a study on heterogeneity within a given cluster. A sensitivity analysis was performed following exclusion of those studies found likely to be influential. Results of both the primary analysis and sensitivity analysis are provided^14^. Analysis was performed using the *mada* package on R version 4.0.0. p values < 0.05, unless otherwise specified, were accepted as significant.

## Results

**Figure 1:**
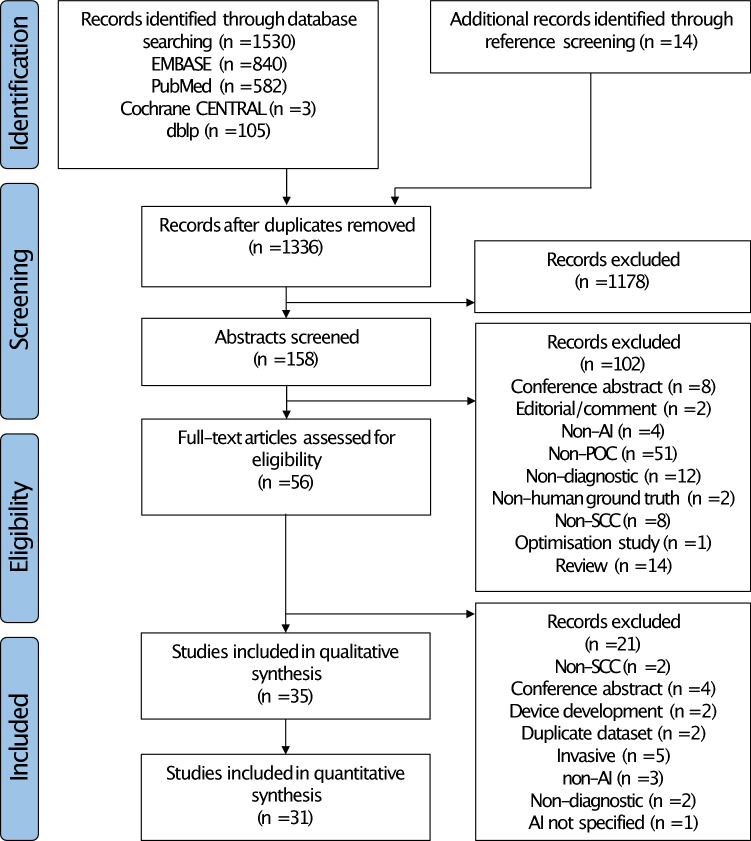
PRISMA flow diagram for study selection.

The initial literature search identified 1530 studies across the 4 databases, and a further 14 studies were identified following iterative review of references (Fig. 1). 1336 studies remained following removal of duplicates. Of these, 35 studies met the inclusion criteria for downstream analysis (Tables 1, 2 and 3). Four of these studies did not report sensitivity and specificity, and were, thus, included in qualitative synthesis only^15–18^.

**Table 1:** Summary of identified studies using clinical photography as the screening modality.

Study	Data source	ML classification methods	Performance metrics	Outcomes (best performing ML)
**Clinical photographs**				
Fu et al.^19^	Heterogenous dataset from both smartphones and SLR cameras	NN based on DenseNet121 architecture, pre-trained on ImageNet	Sensitivity Specificity Accuracy AUROC t-SNE	Sensitivity 89.6 Specificity 80.6 Accuracy 84.1 AUROC 0.935
Welikala et al.^20^	Smartphone images of oral lesions as part of the MeMoSA initiative	NN based on ResNet101 architecture, pre-trained on ImageNet	Sensitivity Precision F_1_	Sensitivity 89.51 Precision 84.77 F_1_ 87.07
Jubair et al.^21^	Heterogenous dataset from both smartphones and SLR cameras	NN based on EfficientNet architecture, pre-trained on ImageNet	Sensitivity Specificity Accuracy AUROC	Sensitivity 86.7 Specificity 84.5 Accuracy 85.0 AUROC 0.928
Shamim et al.^22^	Images extracted directly from search engines	Multiple pre-trained NNs. Best performing algorithm based on VGG19 architecture	Sensitivity Specificity Accuracy AUROC Tsec	Sensitivity 89.0 Specificity 97.0 Accuracy 0.98 AUROC 0.990 212.09 s
Warin et al.^23^	Clinical photography. Specific imaging method not disclosed	NN based on DenseNet121 architecture, pre-trained on ImageNet	Sensitivity Specificity Precision AUROC F_1_ Grad-CAM	Sensitivity 98.75 Specificity 100 Precision 100 AUROC 0.99 F_1_ 0.99
Lin et al.^24^	Heterogenous dataset from 4 different smartphones	NN based on HRNet-W18 architecture, pre-trained on ImageNet	Sensitivity Specificity Precision AUROC F_1_ Grad-CAM	Sensitivity 83.0 Specificity 96.6 Precision 0.84 AUROC 0.946 F_1_ 0.9
Welikala et al.^25^	Smartphone images of oral lesions as part of the MeMoSA initiative	Multiple pre-trained NNs. Best performing algorithm based on VGG19 architecture	Sensitivity Specificity Precision Accuracy F_1_ Grad-CAM	Sensitivity 85.7 Specificity 76.4 Precision 0.77 Accuracy 80.9 F_1_ 0.81
Figueroa et al.^26^	Clinical photographs. Specific imaging method not disclosed	NN based on VGG19 architecture, pre-trained on ImageNet	Sensitivity Specificity Accuracy Grad-CAM	Sensitivity 74.4 Specificity 89.1 Accuracy 83.8
Warin et al.^27^	SLR camera	NN based on ResNet architecture, pre-trained on ImageNet	Sensitivity Specificity Precision AUROC	Sensitivity 98.4 Specificity 91.7 Precision 92.0 AUROC 0.950
Tanriver et al.^28^	Clinical photographs taken in clinical department, supplemented by images from various search engines	Multiple pre-trained NNs; best performance using EfficientNet-b4 architecture	Sensitivity Precision F_1_	Sensitivity 89.3 Precision 86.2 F_1_ 85.7
Jeyaraj et al.^29^	Imaging data extracted from UCI irvine machine learning repository, the cancer imaging archive and the genomic data commons data portal	Modified Inception v3 architecture pre-trained on ImageNet. Compared to support vector machine and deep belief network	Sensitivity Specificity Accuracy AUROC	Sensitivity 98.0 Specificity 94.0 Accuracy 96.6 AUROC 0.965

**Table 2:** Summary of identified studies using optical imaging as the screening modality.

Study	Data source	ML classification methods	Performance metrics	Outcomes (best performing ML)
**Optical imaging**				
Uthoff et al.^30^	Custom smartphone-based dual modality device capable of both white light and autofluorescence imaging	NN based on VGG-M architecture, pre-trained on ImageNet	Sensitivity Specificity Precision NPV Accuracy AUROC	Sensitivity 85.0 Specificity 89.0 Precision 0.88 NPV 0.85 Accuracy 86.9 AUROC 0.91
Song et al.^17^	Smartphone-based intraoral imaging system with custom WL probe	NN based on VGG19 architecture, pre-trained on ImageNet	Accuracy	Accuracy 85.6
Chan et al.^31^	VELscope device^32^	Classification based ResNet or Inception architecture, using either a fully convolutional network or feature pyramid network	Sensitivity Specificity	Sensitivity 98.0 Specificity 88.0
Aubreville et al.^33^	Confocal Laser Endomicroscopy images of oral cavity following IV fluorescein. Images extracted from IO videos. CystoFlex UHD and Coloflex UHD as imaging devices	Used untrained LeNet-5 architecture with patch probability fusion, whole image classification using pre-trained Inception V3 CNN and random forest classifier. Best performance using LeNet-5	Sensitivity Specificity Accuracy AUROC	Sensitivity 86.6 Specificity 90.0 Accuracy 88.3 AUROC 80.7
De Veld et al.^15^	Xe lamp with monochromator for illumination, a spectrograph and custom set of long-pass and short-pass filters	NN with base architecture not specified; single hidden layer between input and output	AUROC	AUROC 0.68
Roblyer et al.^34^	Multispectral digital microscope (MDM), measuring white light reflectance, autofluorescence, narrow band reflectance and cross-polarised light	Linear discriminant analysis	Sensitivity Specificity AUROC	Sensitivity 93.9 Specificity 98.1 AUROC 0.981
Caughlin et al.^35^	Multispectral autofluorescence lifetime imaging (maFLIM) endoscopy	Bespoke neural network using a shared encoder and separate paths for signal reconstruction and classification; classification on pixel-pixel basis	Sensitivity Specificity Precision Accuracy F_1_	Sensitivity 87.5 Specificity 67.6 Precision 76.3 Accuracy 77.6 F_1_ 0.80
Jo et al.^36^	Time-domain multispectral FLIM rigid endoscope. Emission spectral collected for collagen, NADH, FAD	Quadratic discriminant analysis	Sensitivity Specificity AUROC	Sensitivity 95 Specificity 87 AUROC 0.91
Francisco et al.^37^	Portable spectrophotometer with two solid state lasers; a diode emitting at 406 nm and a double frequency neodymium 523 nm as excitation source	Compared naïve bayes, k-Nearest Neighbours and decision tree. Decision tree provided best performance	Sensitivity Specificity Accuracy	Sensitivity 87.0 Specificity 91.2 Accuracy 87.0
Wang et al.^19^	Fibre optics-based flurospectrometer, using Xe lamp with monochromator as excitation source	Partial least squares combined with artificial neural network—neural network with single hidden layer	Sensitivity Specificity Precision	Sensitivity 81.0 Specificity 96.0 Precision 88
Majumder et al.^38^	N_2_ laser as excitation source	Relevance Vector Machine (RVM)	Sensitivity Specificity AUROC	Sensitivity 91 Specificity 95 AUROC 0.9
Huang et al.^39^	VELscope device	Quadratic discriminant analysis	Sensitivity Specificity	Sensitivity 92.3 Specificity 97.9
Duran-Sierra et al.^40^	Multispectral autofluorescence lifetime imaging endoscopy (maFLIM); preferential excitation of NADH and FAD	Best performance using ensemble approach of support vector machine and quadratic discriminant analysis	Sensitivity Specificity F1 AUROC	Sensitivity 94.0 Specificity 74.0 F1 0.85 AUROC 0.81
Jeng et al.^41^	VELscope device	Used both linear discriminant analysis and quadratic discriminant analysis	Sensitivity Precision Accuracy F_1_ AUROC	Sensitivity 92.0 Precision 0.86 Accuracy 86.0 F_1_ 0.88 AUROC 0.96
Huang et al.^42^	Custom autofluorescence device, comprising two LED continuous wave lamps, for preferential imaging of NADH and FAD	Quadratic discriminant analysis	Sensitivity Specificity	Sensitivity 94.6 Specificity 85.7
Kumar et al.^43^	Custom portable autofluorescence device using collimating lens and bream splitter; 405 nm dioxide for excitation	Dimensionality reduction using PCA, before Mahalanobis distance classification on first 11 PCs	Sensitivity Specificity Accuracy	Sensitivity 98.7 Specificity 100 Accuracy 98.9
Rahman et al.^44^	Custom portable imaging system composed of modified headlamp system capable of both autofluorescence imaging and reflectance imaging	Linear discriminant analysis	Sensitivity Specificity AUROC	Sensitivity 92.0 Specificity 84.0 AUROC 0.913
James et al.^45^	Use of a spectral-domain Optical Coherence Tomography (OCT) system consisting of a 2D scanning long GRID rod probe with a centre wavelength of 930 nm	Use of 14 artificial neural networks for feature extraction, followed by support vector machine for classification. Best performance using DenseNet-201 and NASNetMobile in delineating OSCC from others	Sensitivity Specificity PPV NPV Accuracy	Sensitivity 86.0 Specificity 81.0 PPV 51.0 NPV 96.0 Accuracy 81.9

**Table 3:** Summary of identified studies using thermal imaging and VOC analysis as the screening modality.

Study	Data source	ML classification methods	Performance metrics	Outcomes (best performing ML)
**Thermal imaging**				
Chakraborty et al.^16^	FLIR T 650 SC long infrared (7.5–13 µm) camera	Support Vector Machine (SVM)	Accuracy	Accuracy 84.72
**Detection of volatile organic compounds (VOCs)**				
Van de Goor et al.^46^	‘Aeonose’ electronic nose—using 3 micro-hotplate metal-oxide sensors to detect a range of VOCs in exhaled breath	Compression of 64 × 36 measurements per sensor, using Tensor Decompression (Tucker3-like). NN implemented through AeoNose software (Aethena software)—base architecture not specified	Sensitivity Specificity Accuracy AUROC	Sensitivity 84 Specificity 67 Accuracy 72 AUROC 0.850
Mohamed et al.^47^	‘Aeonose’ electronic nose—using 3 micro-hotplate metal-oxide sensors to detect a range of VOCs in exhaled breath	Compression of 64 × 36 measurements per sensor, using Tensor Decompression (Tucker3-like). NN implemented through AeoNose software (Aethena software)—base architecture not specified	Sensitivity Specificity Precision Accuracy AUROC	Sensitivity 80 Specificity 77 Precision 67 Accuracy 79 AUROC 0.882
Leunis et al.^48^	‘DiagNose’ electronic nose—12 metal-oxide sensors using four different sensor types: CH_4_, CO, NO_x_, Pt	Forward selection logistic regression	Sensitivity Specificity AUROC	Sensitivity 90 Specificity 80 AUROC 0.850
Hakim et al.^49^	‘Nanoscale Artificial Nose’ (NA-NOSE) electronic nose—5 sensors based on gold nanospheres with tert-dodecanethiol, hexanathiol, 2-mercaptobenzoazole, 1-butanethiol, and 3-methyl-1-butanethiol ligands	Support vector machine (SVM) trains on principle components 1 and 2, following PCA of sensor measurements	Sensitivity Specificity Accuracy	Sensitivity 100 Specificity 92 Accuracy 96
Mentel et al.^18^	‘BreathSpect’ device, utilising two fold separation using gas chromatography and mass spectrometry to detect VOCs	2-Dimensional output from ‘BreathSpect’ device converted to integer arrays. Best classification performance using logistic regression	Accuracy	Accuracy 89

**Figure 2:**
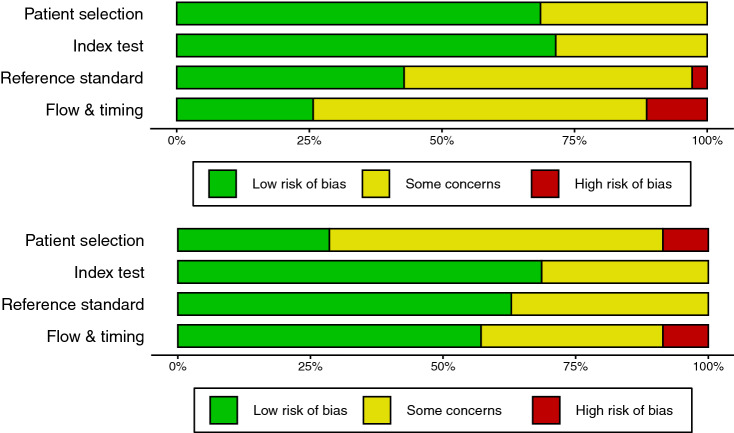
Summary plots of ‘Risk of bias’ (top panel) and ‘Applicability’ (bottom panel) using the QUADAS-2 tool.

The results of the QUADAS-2 tool are provided in Fig. 2 and Supplemental Fig. S1. Eight studies were found to have a high risk of bias across any of the 7 domains^2,16,21,22,26,28,30,35^. Within domain 1, 11% of studies were found to have high risk of bias, 26% low risk of bias, and 63% unclear risk of bias. Within domain 2, just 1 study was found to have high risk of bias, 43% low risk and 54% unclear risk. Within domain 3, 71% studies were found to have a low risk of bias and 29% with unclear risk. In domain 4, 69% had low risk and 31% had unclear risk of bias.

Four broad categories of methodologies were identified in POC detection of oral potentially malignant and malignant disorders: (1) classification based on clinical photographs (n = 11)^2,19–23,25–29^; (2) in vivo imaging using intra-oral optical imaging techniques (n = 18)^15,17,30,31,33–35,37–45,50^; (3) thermal imaging (n = 1)^16^; (4) analysis of volatile organic compounds (VOCs) from breath samples (n = 5)^18,46–49^. Just 8 studies were published before 2015^15,34,37,38,44,48–50^. The majority of studies provided data on classification of OSCC vs healthy (n = 13)^16,18,19,23,31,33,38,42,43,46–49^, 8 studies provided data on OSCC/OPMD vs healthy^25,26,28,30,37,39–41^, 6 on OSCC/OPMD vs benign lesions^15,17,21,35,36,50^, 3 on OSCC vs benign^29,34,44^, 2 on OSCC vs other (healthy, benign and OPMD)^2,45^, 1 on OSCC/OPMD vs benign/healthy^20^, 1 on OPMD vs healthy^27^, and 1 on OPMD vs benign^22^.

**Figure 3:**
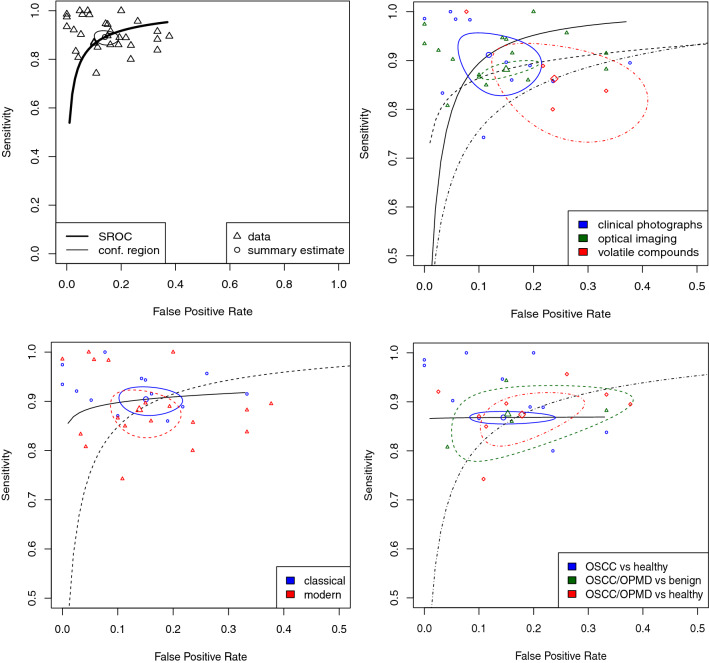
Summary Receiver Operator Characteristic (sROC) curves to estimate model performance; Top left, sROC curve of bivariate model of all studies (AUC 0.935); top right, sROC curves according to methodology; bottom left, sROC curves according to AI type; bottom right, sROC curves according to lesion type. AUC for subgroups, and results of subgroup analysis are provided in Table 4.

Given sample heterogeneity, as indicated by forest plots (Supplementary Fig. S2) of univariate meta-analyses and quantitative measures of heterogeneity (sensitivity: Tau^2^ = 0.37, *I*^2^ = 62%, p < 0.001; specificity: Tau^2^ = 0.70, *I*^2^ = 84%, p < 0.001), a bivariate random-effects model for logit-transformed pairs of sensitivities and false positive rates was used to provide an estimate of diagnostic test performance. Across all studies, the pooled estimates for sensitivity and false positive rates (FPR) were 0.892 [95% CI 0.866–0.913] and 0.140 [95% CI 0.108–0.180], respectively. The AUC was 0.935 (partial AUC restricted to observed FPRs of 0.877), indicating excellent classifier performance (Table 4; Fig. 3, top left panel).

**Table 4:** Results of main bivariate random effects model of diagnostic test performance, subgroup analysis, and sensitivity analysis following removal of influential outliers.

Category	Subgroup	Sensitivity [95% CI]	False positive rate [95% CI]	AUC [restricted AUC]	Diagnostic meta-regression estimate (SE); p-value	
					Sensitivity	False positive rate
**Main analysis**						
Overall	–	0.892 [0.866; 0.913]	0.140 [0.108; 0.180]	0.935 [0.877]	–	–
AI type	Classical	0.904 [0.878; 0.925]	0.151 [0.111; 0.202]	0.915 [0.893]	–	–
	Modern	0.883 [0.839; 0.916]	0.139 [0.096; 0.197]	0.932 [0.867]	− 0.341 (0.247), p = 0.167	− 0.003 (0.320), p = 0.994
Modality	Volatile compounds	0.863 [0.764; 0.924]	0.238 [0.142; 0.372]	0.889 [0.827]	–	–
	Clinical photographs	0.911 [0.848; 0.950]	0.118 [0.070; 0.192]	0.952 [0.900]	0.401 (0.464), p = 0.388	− 0.740 (0.490), p = 0.131
	Optical imaging	0.882 [0.865; 0.896]	0.150 [0.112; 0.197]	0.914 [0.867]	0.328 (0.450), p = 0.131	− 0.620 (0.476), p = 0.192
Lesion type	OSCC vs healthy	0.868 [0.858; 0.878]	0.145 [0.093; 0.218]	0.861 [0.859]	–	–
	OSCC/OPMD vs benign	0.875 [0.801; 0.924]	0.153 [0.063; 0.326]	0.905 [0.869]	− 0.222 (0.342), p = 0.516	0.122 (0.490), p = 0.803
	OSCC/OPMD vs healthy	0.874 [0.824; 0.911]	0.179 [0.115; 0.268]	0.914 [0.852]	0.205 (0.385), p = 0.594	0.205 (0.385), p = 0.594
**Sensitivity analysis (influential outliers removed)**^**a**^						
Overall	–	0.892 [0.871; 0.910]	0.142 [0.104; 0.190]	0.883 [0.883]	–	–
AI type	Classical	0.903 [0.875; 0.924]	0.176 [0.150; 0.205]	0.931 [0.867]	–	–
	Modern	0.878 [0.843; 0.907]	0.118 [0.068; 0.199]	0.870 [0.870]	− 0.248 (0.207), p = 0.232	− 0.349 (0.362), p = 0.335
Modality	Volatile compounds	0.921 [0.863; 0.856]	0.157 [0.124; 0.197]	0.916 [0.912]	–	–
	Clinical photographs	0.899 [0.861; 0.928]	0.084 [0.041; 0.168]	0.920 [0.890]	0.244 (0.433), p = 0.574	− 0.784 (0.583), p = 0.179
	Optical imaging	0.896 [0.868; 0.238]	0.172 [0.122; 0.238]	0.904 [0.884]	0.275 (0.419), p = 0.512	− 0.127 (0.547), p = 0.817
Lesion type	OSCC vs healthy	0.900 [0.861; 0.929]	0.185 [0.149; 0.227]	0.919 [0.866]	–	–
	OSCC/OPMD vs benign	0.875 [0.801; 0.924]	0.152 [0.063; 0.326]	0.905 [0.869]	− 0.347 (0.306), p = 0.256	0.002 (0.479), p = 0.997
	OSCC/OPMD vs healthy	0.904 [0.863; 0.934]	0.168 [0.087; 0.299]	0.910 [0.894]	− 0.070 (0.275), p = 0.256	0.083 (0.464), p = 0.858

^a^Influential studies removed for sensitivity analysis^2,20,25,26,30,33,38,43,46^.

Graphic Display of Study Heterogeneity (GOSH) plots were used to further explore causes of heterogeneity in the extracted data through the application of unsupervised clustering algorithms to identify influential outliers (Supplemental Fig. S3). 4 studies were found to substantially contribute to between-studies heterogeneity with respect to sensitivity^27,28,33,40^, and a further 6 studies were identified as potentially influential with respect to specificity^20,24,25,33,38,43,46^. Exclusion of these studies from a univariate random effects model of sensitivity (N = 27) and specificity (N = 24) resulted in a reduction in Higgins *I*^2^ to 0.0% [0.0; 42.5] (Tau^2^ = 0.27, Q(26) = 24.99, p = 0.52) for sensitivity and *I*^2^ 60.8% [38.9; 74.8] (Tau^2^ = 0.39, Q(23) = 58.7, p < 0.0001). A sensitivity analysis was thus performed with influential outliers excluded (Table 4). Although these analyses provide an indication of influential outlying studies, they do not inform on the likelihood of small study effects as a contributor of identified heterogeneity.

Funnel plots, of both all studies and according to subgroup, were initially used to investigate for small study effects (Supplemental Fig. S4). These funnel plots themselves provide an indication of possible publication bias, with a number of studies demonstrating both a large effect size and standard error, and the use of contour-enhancement does appear to identify a scarcity of studies in zones of low significance. Egger’s linear regression test supported plot asymmetry within studies reporting on classical machine learning methods (Supplemental Table S2). These results should be interpreted with caution, however, and plot asymmetry alone is not pathognomonic of publication bias. To further investigate small study effects as a possible cause for this asymmetry, a bias-corrected estimate of the diagnostic odds ratio was determined using Duval and Tweedie’s Trim and Fill method, which aims to re-establish symmetry of the funnel plot by imputing ‘missing’ effects, to provide an adjusted diagnostic odds ratio that better reflects the true effect when all evidence is considered. This method did identify a reduction in effect size, particularly in studies reporting on classical machine learning methods in classification, in those examining the use of clinical photographs, and in those classifying OSCC vs Healthy. Inspection of the funnel plots for these categories (Supplemental Fig. S4) does appear to show an absence of studies within regions of low significance, supporting a conclusion that reporting bias may contribute to inflation of study effects in some subgroups.

A comparison of algorithm performance according to methodology (clinical photographs, thermal imaging or analysis of volatile compounds), AI type (modern and classical), and lesion type (OSCC vs Healthy, OSCC/OPMD vs Benign, OSCC/OPMD vs Healthy) identified no differences in performance, as indicated by overlap in confidence regions on sROC curves (Fig. 3), showing uniformly high performance irrespective of group. Moreover, bivariate meta-regression found no significant differences in classification performance irrespective of methodology, AI type or lesion type (Table 4). A comparison of lesion types undergoing classification was limited to OSCC vs Healthy, OSCC/OPMD vs Benign, OSCC/OPMD vs Healthy, given the limited number of studies reporting results on other comparisons. Classification performance across subgroups was similar following exclusion of those studies identified as potentially influential.

Just 1 study met the inclusion criteria reporting on the use of thermal imaging in oral cancer detection^16^. In this study, Chakraborty et al. exploited Digital Infrared Thermal Imaging (DITI) as a non-invasive screening modality for oral cancer. Their process of detection involves initial detection of left and right regions of interest (ROI) from infrared images using a FLIR T 650 SC long infrared camera. Rotation invariant feature extraction was then performed on ROI using a Gabor filter, the responses of which are then used as input into a non-linear support vector machine (SVM) following transformation using a radial basis function (RBF) kernel. Fivefold cross validation on a dataset of 81 malignant, 59 precancerous and 63 normal subjects identified an overall accuracy of 84.72% in distinguishing between normal vs malignant subjects.

18 studies used various methods of optical imaging for *in-vivo* detection of oral potentially malignant and malignant disorders^15,30,31,33–45,50,51^, 16 of which provided sufficient performance metrics for meta-analysis^15^. All studies were prospective in design. Estimates for sensitivity and false positive rate for this modality were 0.882 [95% CI 0.865–0.896] and 0.118 [0.112–0.197], respectively. AUC for the accompanying sROC curve (Fig. 3) was 0.914 (partial AUC of 0.867); again, indicating good classifier performance. The majority of studies exploited perturbation in autofluorescence spectra in oral pathology as the principal method of detection. However, there was variation in the source and wavelengths of excitation (Table 2). With exception to 11 studies (which used a support vector machine^40,45^, relevance vector machine^38^, quadratic discriminant analysis^36,39,41,42^, Mahalanobis distance^43^, linear discriminant analysis^34,52^, and decision tree^37^), the remaining studies demonstrated best performance using neural networks. In studies utilising ANN, data pre-processing was similar, involving some form of normalisation to standardise contrast and brightness, before introduction of a size-adjusted image according to the base architecture (Supplementary Data S1). The exceptions here were Chan et al., who instead utilised a Gabor filter or wavelet transformation from a redox ratio image of FAD and NADH to ultimately generate a feature map as input, Wang et al., who used partial least squares discriminant analysis on captured spectra to identify features as input, and de Veld et al. who again utilised normalised autofluorescence spectra as input. 3 studies used augmentation to increase the size of the training dataset for ANN^30,33,51^. Contrarily, studies utilising classical ML techniques for classification were heavily reliant on manual region of interest (ROI) detection and manual feature extraction. All studies with exception to James et al. produced a series of spectral intensity-based features following normalisation as input for classification. James et al. instead adopted an ensemble approach, whereby object detection and feature extraction were automated using ANNs, before introduction into a support vector machine for classification. Best overall accuracy within the modern ML group was achieved by Chan et al. using Inception (accuracy of 93.3) to classify OSCC vs healthy, and best performance within the classic group was achieved by Kumar et al. (accuracy 99.3) using Mahalanobis distance in classification of OSCC vs healthy.

Uthoff et al. performed a field-testing study of new hardware developed specifically for intra-oral classification of benign and (pre-)malignant lesions. The device in question, designed to provide POC detection in low- and middle-income countries, comprises an intra-oral probe connecting to a standard widely available smartphone that utilises 6 405 nm LEDs for autofluorescence and 4 4000 K LEDs for white light. Classification of autofluorescence spectra using a VGG-M architecture provided an accuracy of 86.88%, and AUC of 0.908. Song et al. also used a custom smartphone-based intra-oral visualisation system, exploiting 6 405 nm LEDs for excitation. This approach, using a VGG-M architecture pretrained on ImageNet, yielded an accuracy of 86.9%, with sensitivity of 85.0% and specificity of 88.7%^51^. Other approaches for achieving autofluorescence in vivo included a xenon lamp with monochromator and spectrograph^15^, multispectral digital microscopy^35^, time-domain multispectral endogenous fluorescence lifetime imaging FLIM^36^, N_2_ laser^38^, confocal endomicroscopy (CFE)^33^, portable spectrophotometry^37,50^, and optical coherence tomography^45^. Notably, although in vivo and providing a prospect of POC detection, the approach taken by Aubreville et al. of confocal laser endomicroscopy does require intra-venous administration of fluorescein prior to imaging and its utility as a POC detection tool may therefore be limited^33^. Both Huang et al. and Jeng et al. used the commercially available VELscope for autofluorescence imaging, though both groups used different approaches to classification. Huang et al. determined the average intensity of red, blue and green (RGB) channels and grayscale following grayscale conversion as input into quadratic discriminant analysis to distinguish between oral potentially malignant/malignant and healthy tissues, reporting a sensitivity and specificity of 0.92 and 0.98, respectively^39^. While feature selection was similar to Huang’s group (extracting average intensity and standard deviation of intensity from grayscale-converted RGB images), Jeng et al. compared the performance of both linear discriminant analysis (LDA) and quadratic discriminant analysis (QDA), reporting an optimal performance using QDA on normalised images of the tongue (sensitivity of 0.92, precision 0.86)^41^.

11 of the 26 identified studies attempted diagnosis of oral potentially malignant or malignant disorders from clinical photographs^19–29^, all of which utilised deep learning through various neural network architectures for classification and were retrospective in design (Table 1). All studies using clinical photographs provided performance metrics amenable to meta-analysis. Sensitivity and false positive rate were estimated as 0.911 [95% CI 0.848–0.950] and 0.118 [95%CI 0.070–0.192], respectively, and AUROC was 0.952 (partial AUC of 0.90; Fig. 3). All studies in this category used neural networks for classification. The source of images was variable between studies, with 4 studies using smart phone cameras as a potential easily-implementable POC source of data^20,24–26^, 2 studies using heterogenous images from various camera types^19,21^, 3 studies using images from search engines/repositories^22,28,29^, and 2 used high resolution single-lens reflex (SLR) cameras^23,27^. Training and testing sample sizes varied between studies (Fig. 5), though 8 of the 11 studies used augmentation to enhance the size of the training set, including scaling, shearing, rotation, reflection, and translation^19,20,23–28^. With exception to Fu et al. (who used the Single Shot Multibox Detector (SDD) as a detection network), and Lin et al.^24^ (who used the automatic centre-cropping function of a smartphone grid), all remaining studies within this category depended upon manual ROI bounding, thus still requiring a degree of clinical expertise prior to feature extraction and classification. Best overall accuracy, of 99.28, was achieved by Warin et al.^23^ using DenseNet-161 (pretrained on ImageNet) in classification of OSCC from healthy.

Fu et al. developed a two-stage process of classification, exploiting the Single Shot MultiBox Detector (SSD) as a detection convolutional neural network to initially define the region of interest, before binary classification using DenseNet, pretrained on ImageNet. In addition to demonstrating promising classification performance (AUROC 0.970), the developed deep learning algorithm also demonstrated superior performance in classification from clinical images compared to blinded non-medical professionals and post-graduate medical students majoring in oral and maxillofacial surgery (OMFS). Both identified studies by Welikala et al. adopted a smart phone-based approach, with a view to rapid POC detection of oral cancer in low and middle-income countries, as part of the Mobile Mouth Screening Anywhere (MeMoSA) initiative. A range of convolutional neural networks were trained on provided images, with best classification performance achieved through the VGG-19 architecture (Table 1). Both Tanriver et al. and Jeyaraj et al. attempted multiclass classification of either OSCC vs OPMD vs benign or normal vs benign vs malignant, respectively. Both used search engines and existing data repositories as the source of input data for classification (though Tanriver supplemented these using clinical photography within their unit). Transfer learning, with pretraining on ImageNet, performed best using the EfficientNet-b4 architecture in Tanriver et al., reporting an F_1_ of 0.86. Jeyaraj modified the Inception v3 architecture, and compared to a support vector machine and deep belief network, reporting a specificity of 0.98 and sensitivity of 0.94.

4 studies provided data on the use of an electronic nose as a POC device to detect malignancy-associated volatile compounds from exhaled breath (Table 3), all with exception to Mentel et al. providing outcomes amenable to meta-analysis^46–49^. All studies were prospective in design. Pooled estimates for sensitivity and false positive rate were 0.863 [95% CI 0.764–0.924] and 0.238 [95% CI 0.142–0.372] and AUC was estimated at 0.889 (partial AUC of 0.827). All 4 studies utilised some form of portable electronic ‘nose’ (eNose) to detect volatile organic compounds in exhaled breath of either patients with a confirmed diagnosis of malignancy or healthy controls. Van der Goor et al. and Mohamed et al. used eNose devices with a combination of micro hotplate metal-oxide sensors to detect changes in conductivity with redox reactions of volatile organic compounds on heating. Leunis instead analysed air samples using 4 sensor types—CH_4_, CO, NO_x_ and Pt—and Hakim et al. used a device dependent upon spherical gold nanoparticles. Van der Goor et al. and Mohamed et al. both used tensor decomposition (Tucker3) to generate a single input vector for training of a neural network from the 64 × 36 datapoints generated per sensor, achieving sensitivities of 84% and 80%, and specificities 80% and 77% in detecting OSCC. Leunis et al. instead used logistic regression in binary classification, using measurements from only the NO_x_ sensor to avoid collinearity. This achieved a sensitivity of 90% and specificity of 80%. Hakim et al. used Principal Component Analysis (PCA) for initial clustering, before training a linear support vector machine on principle components 1 and 2—this method achieved a sensitivity of 100% and specificity of 92%. Mental et al. used a commercially available BreathSpect device for sample collection, using two-fold separation with gas chromatography and mass spectrometry to detect VOCs. The output from the affiliated software is a 2-dimensional image representation of both VOC drift time and parts-per-billion. This output was used to train various classical machine learning algorithms (k-nearest neighbours, random forest, logistic regression and linear discriminant analysis), with best performance of an accuracy of 0.89 using logistic regression.

**Figure 4:**
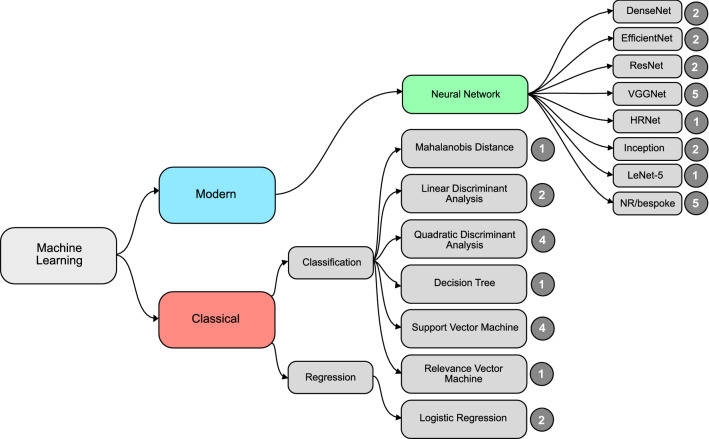
Summary of best performing machine learning algorithms adopted by identified studies. The numbers represent the number of studies who reported best outcomes with the associated model. *VGG* visual geometry group, *HR* high resolution, *NR* not reported.

Several approaches to ML were used across the identified studies in their pursuit for detection of oral potentially malignant and malignant disorders. For clarity, the hierarchical classification presented by Mahmood et al. is adopted here^53^. ML classification algorithms may be subdivided into modern techniques and classical techniques (Fig. 4). The majority of identified studies used supervised algorithms for classification (following feature selection where necessary), whereby the machine is trained on annotated data. The majority of studies reported best outcomes using various architectures of neural networks. All studies on analysis of photographic images used deep learning (neural networks with more than one hidden layer), the most popular architecture of which being VGG neural networks^17,22,25,26,30,51^. This is perhaps unsurprising since VGGNet was developed as an extension of the revolutionary AlexNet^54,55^.

Several studies compared multiple different machine learning methods in classification. Shamim et al. used transfer learning with multiple convolutional neural networks pretrained on ImageNet, including AlexNet, GoogLeNet, VGG19, ResNet50, Inception v3 and SqueezeNet, achieving the optimal performance using the VGG19 CNN with a sensitivity of 89% and specificity of 97%^22^. Welikala et al. compared VGG16, VGG19, Inception v3, ResNet50 and ResNet101, all pretrained on ImageNet and applied through transfer learning; VGG19 again proved to provide the best detection of suspicious lesions from clinical images. Tanriver et al. found optimal performance using the EfficientNet-b4 architecture in clinical image classification.

Fifteen studies used “classical” ML algorithms. Roblyer et al. and Rahman et al. used linear discriminant analysis for classification of features extracted from autofluorescence images. Jo et al. and Huang et al. used quadratic discriminant analysis. Duran-Sierra et al. exploited an ensemble approach of both quadratic discriminant analysis and a support vector machine, demonstrating superior performance in classification of normalised ratios from autofluorescence images than the two approaches independently. Francisco et al. used decision trees, Chakraborty et al. and Hakim et al. used support vector machines, Majumder et al. a relevance vector machine and Leunis et al. used logistic regression. James et al. also adopted an ensemble approach, employing ANN for feature extraction prior to a support vector machine for classification. Feature selection and reduction for input into classical machine learning algorithms was also achieved through a variety of methods, including Principle Component Analysis^49^, tensor decomposition^46,47^, Gabor feature extraction and discrete wavelet transformation^31^. The only study utilising an unsupervised machine learning approach for classification (rather than feature selection) was Kumar et al., who initially used PCA for dimensionality reduction before Mahalanobis distance classification of the first 11 identified principal components.

**Figure 5:**
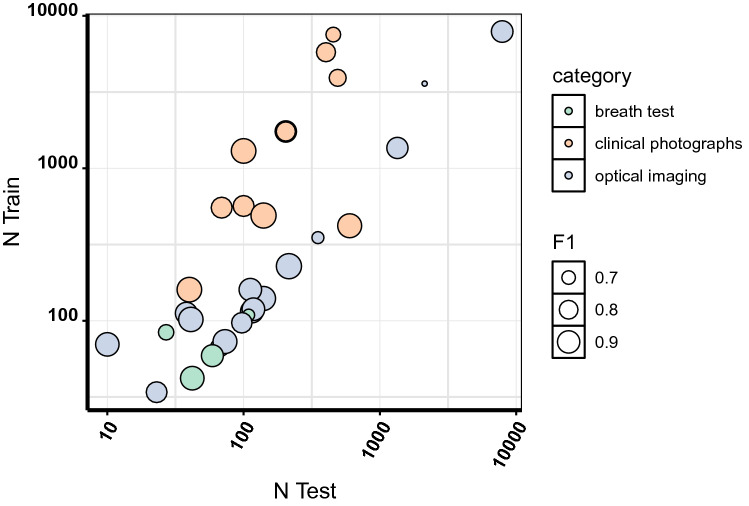
overview of training and validation sample sizes for identified studies included in meta-analysis. Point size proportional to F_1_ score, indicating no obvious relationship between size of training sample here and performance.

Sample sizes for training and validation sets were hugely variable between studies. Test set sample size ranged from 5 per sample^31^ to 4079^33^. An overview of training and test set sample sizes is provided in Fig. 5. Training sample sizes are estimates only, as some papers did not report total sample size post-augmentation, and so only the initial training sample size was recorded (and may therefore be underestimated). 16 of the 35 included studies did not report on software for implementation of machine learning methods. Of those using modern ML methods, 7 studies used the Keras application programming interface^20,21,23,25,27,33,35^, 2 used PyTorch, 1 used the Python Scikit-learn machine learning library, 2 studies used proprietary software accompanying the eNose^46,47^, and 1 study used the Deep Learning Toolbox and Parallel Learning Toolbox within MATLAB^22^. Within studies using classical ML methods, 3 studies used MATLAB^34,43,45^, 1 used Scikit-learn (Python), 1 used SPSS Statistics^48^, and 1 study used WEKA^37^.

## Discussion

Artificial Intelligence is becoming increasingly pervasive in the domains of medical diagnostics and prognostication, afforded by increasingly complex deep learning algorithms that overcome the limitation of manual feature extraction. The realisation that a deep learning algorithm could outperform consultant radiologists in the diagnosis of lung cancer in 2019 certainly instils a sense of cautious optimism that machine learning may provide a feasible solution for automatic cancer detection^56^. The use of machine learning, however, in translational medicine is not limited to radiology. Recent developments have allowed prediction of pharmacological properties of compounds to enhance drug discovery^57^, selection of chemotherapy dose regimes^58^, and prediction of splice variants and transcriptional regulatory mechanisms based on genomics data^59^. This same level of success has unfortunately yet to be translated to head and neck cancer. The purpose of the current study was to provide an update on the progress of machine learning in POC testing for potentially malignant and malignant disorders of the oral cavity.

Thirty-five studies were identified during the literature review, encompassing 4 categories of testing modalities: (1) assessment of clinical photos; (2) analysis of autofluorescence spectra; (3) detection of volatile organic compounds in exhaled air; and (4) thermal imaging. The overall estimates for sensitivity and false positive rates for included studies were 0.892 and 0.140, with an AUC of 0.935. These outcomes suggest good classification performance. Fu et al. undertook an additional analysis, testing their neural network on intra-oral photographs against blinded human performance with varying expertise. It was found that, on a clinical validation dataset of 666 images, the algorithm emphatically outperformed a student panel majoring in OMFS and a panel of non-medical students, and was fairly equivalent in its performance with a panel of oral cancer experts (model accuracy of 92.3% compared to expert accuracy of 92.4%), demonstrating the potential of this technique. No differences were identified between testing modality, AI type or lesion type with respect to diagnostic test performance.

The true potential in the automatic feature selection and classification from intra-oral white light images is that no additional resources, beyond a smartphone and access to an imaging server, are required for POC testing, making this modality particularly appealing for screening in low and middle-income countries. The development of the Mobile Mouth Screening Anywhere (MeMoSA) phone application by Haron et al.^60^, provides an interface between community-based practitioners (usually a general dental practitioner) and specialists, potentially providing a POC platform for machine-learning automated diagnosis^60^. However, there remain limitations with this modality with respect to automation. Many studies using clinical photographs still relied upon the expertise of an oral and maxillofacial expert for delineation of ROI prior to input into a neural network. Arguably, this is still a considerably less resource-intensive exercise than manual classification, and Fu et al. have demonstrated that automated boundary box generation is possible without the need for manual human image annotation. The Visual Geometry Group Networks (VGGNet) proved particularly effective in classification from images where multiple base architectures were compared. VGGNet, as a derivative of AlexNet, provides several additional features to both improve classification performance and computational efficiency^55^. The receptive fields are considerably smaller than that of previous architectures, and the introduction of 3 rectified linear activating function (ReLU) units allows for more robust discrimination.

In contrast to white-light intra-oral imaging, multispectral optical imaging aims to increase visual contrast between non-neoplastic and neoplastic tissue. Autofluorescence spectroscopy has shown promising results in the detection of cancer in a number of other locations, including the lung, oesophagus and colon^61,62^. Tissues contain many fluorophores that re-emit light at specific wavelengths following excitation. Examples of such fluorophores include NADH, FAD, tryptophan, tyrosine and collagen^50^. Alterations in tissue architecture and the distribution of these fluorophores results in a measurable difference in emissions spectra between healthy and neoplastic tissue, providing the basis for the use of tissue autofluorescence as a possible classification method. Studies based on this method also showed promising performance, with an estimated AUC of 0.91. However, de Veld et al., while demonstrating good classification between neoplastic and healthy tissue, did report poor performance of autofluorescence in distinguishing between potentially malignant and malignant disorders relative to Wang et al., which raises a question of generalisability of this technique between populations^15^. A number of commercial devices are currently available that rely on the principle of tissue autofluorescence in detection of oral lesions, showing variable performance across primary studies. These have been comprehensively reviewed previously by Mascitti et al.^63^.

The use of thermal imaging in detection of neoplasia is premised on differences in temperature distribution between potentially malignant, malignant and healthy tissue. The use of Digital Infrared Thermal Imaging (DITI) has previously shown promise as a non-invasive modality for classification of breast and thyroid disease^64,65^. Representing thermal regions of interest as rotation-invariant multiresolution Gabor filter bank responses allowed the input of image-based data into a classical machine learning algorithm in Chakraborty et al., demonstrating good classification performance with a RBF kernelized SVM. The rationale here for introducing a pre-processing stage (Gabor filter) for feature selection with a classical machine learning technique is unclear, particularly given that deep learning architectures optimised for automatic image-based feature selection were available at the time of study (AlexNet for example). This perhaps reflects an insufficient pool of available infrared images for training a deep learning network, and a modern approach to machine learning using DITI certainly warrants further investigation.

The emergence of electronic noses as a means of measuring and analysing volatile compounds in exhaled air has accompanied advances in sensor technologies^47^. Cancer-related VOCs are derived as by-products of cancer metabolism, with different cancers displaying a unique signature of VOCs within various bodily compartments^66^. These VOCs are detectable in exhaled air following diffusion from the blood into the alveoli. This approach also demonstrated good classification performance across the four identified studies, with an AUC of 0.89.

Although subgroup analysis across all studies identified no significant difference in diagnostic test performance between classical and modern classification methods (AUC 0.915 vs AUC 0.932, respectively, p = 0.994), a greater resolution comparison of these methods within lesion type and modality was not possible given the limited number of studies within these subgroups (indeed, classification within the clinical photograph modality was achieved using only ANN). Thus, while it may be true that overall performance is not different across the entire cohort of studies, this does not exclude the possibility of differences in performance between modern and classical classification methods according to specific classification task and the employed diagnostic test. There are potentially sound justifications for why certain ML types were employed in the various studied classification tasks, according to the complexity and amount of data generated through the detection method. Classical approaches require an initial step of feature extraction and, although algorithms exist for automatic feature extraction from images (such as edge detection, corner detection and threshold segmentation), it is still the responsibility of the investigator to decide which features are considered important and which to input into classification. End-to-end learning, through the introduction of a pre-processed image to an ANN, ameliorates this need for intensive tuning and manual feature selection^67^. The major disadvantage here is the computational demand of deep learning. Within optical imaging and breath testing, 9 studies utilised ANN and 14 used classical ML techniques, with no obvious difference in overall diagnostic accuracy according to approach. This is perhaps unsurprising. Where manual feature extraction is not overly cumbersome (and features can be generated from spectral data with relative ease), and training datasets are comparatively small, classical ML techniques may outperform deep learning and avoids the need for big training data and expensive hardware.

Several issues were common to many of the identified studies. Many studies reported performance metrics from internal validation, rather than testing on a discrete external test set to which the algorithm is naïve. Presumably, internal validation only was performed as a means of optimising the amount of available data for training. However, even with very large datasets, the absence of a discrete test/validation set results in overfitting and poor generalisability to the population at large; that is, the trained algorithm functions only in the narrow context within which it is developed^68^. This does present issues where algorithms are trained on homogenous samples, but where substantial heterogeneity is seen in real-world applications, and machine learning algorithms will need to demonstrate sound generalisability before widespread adoption as mainstream diagnostic adjuncts. Heterogeneity was identified as high throughout univariate analysis of both sensitivity and specificity. A sensitivity analysis, excluding influential outlying studies, did support similar results to the main analysis. However, interrogation of small study effects did identify a high likelihood of publication bias, particularly in some subgroups, and a bias-adjusted model found that diagnostic performance was likely over-estimated. Further, a number of studies were ranked as ‘unclear’ across many of the domains of bias and applicability using the QUADAS-2 tool (Fig. 2 and Supplemental Fig. S1). Across many studies, the methods sections simply provided insufficient information to facilitate a reasonable determination of risk of bias.

There are several limitations of the current study. As with any systematic review, there is always potential for the search process to miss relevant articles, providing an incomplete summary of the topic of interest. A particular issue here common to search strategies on automated classification is that classical approaches are often not explicitly referred to as machine learning (or similar such key terms). A highly sensitive search strategy, with a thorough iterative approach to reference screening, was used to mitigate this limitation.

For a machine learning algorithm to be useful as screening tool, it is not necessary to achieve an equivalent accuracy to expert diagnosis. Consider the conventional Papanicolaou (Pap) smear as an example. This screening tool, for cervical intra-epithelial neoplasia, has a sensitivity of 51% and a specificity of 66.6%^69^, but was immensely successful in reducing incidence of cervical squamous cell carcinoma prior to its supercedence by HPV detection. The current difficulty with detection of potentially malignant and early malignant disorders of the oral cavity is the need for expert interpretation of biopsy, a process that is both invasive and time-intensive. Any method that is easily implementable and has a sufficient negative-predictive value to exclude non-cases effectively and safely will be beneficial, and machine learning has the potential to fill this void.

Increasingly deep neural networks, concomitant with advances in computational power and algorithm efficiency, provide opportunity for automated feature selection from complex data. These advancements have translated to a number of promising screening methods for detection of oral potentially malignant and malignant disorders, including detection from clinical photographs, autofluorescence images and exhaled breath samples. The results of the current study provide evidence of reliable lesion classification using these methods, many of which provide opportunity for POC screening in low and middle-income countries lacking expert support and specialist equipment. Further interrogation of the discussed machine learning implementations in heterogenous sample populations is necessary to confirm generalisability.

## Supplementary Information

Supplementary Information 1.

Supplementary Information 2.

Supplementary Information 3.

## Data Availability

All scripts used for data analyses are available upon request from the corresponding author.
