# Supplementary material for: Machine learning in point-of-care automated classification of oral potentially malignant and malignant disorders: a systematic review and meta-analysis
Source: Sci Rep. 2022 Aug 13;12:13797. doi: 10.1038/s41598-022-17489-1 (PMC9376104; doi:10.1038/s41598-022-17489-1)
Supplement: Supplementary file 3 — Supplementary Information 3. [file 41598_2022_17489_MOESM3_ESM.pdf]

Title Page

Article Title: **Machine Learning in Point-of-Care Automated Classification of Oral Potentially Malignant and Malignant Disorders: A Systematic Review and Meta-analysis**

Author List: Ashley **Ferro**[1,2]; Sanjeev **Kotecha**[1,2]; Kathleen **Fan**[1,2]*

Author Affiliations:

1. Faculty of Dentistry, Oral and Craniofacial Sciences, King's College London, United Kingdom
2. Oral and Maxillofacial Surgery Department, King's College Hospital NHS Foundation Trust, Denmark Hill, SE1 9RT, London, United Kingdom

Corresponding Author*
Professor Kathleen Fan
Oral and Maxillofacial Surgery Department,
King's College Hospital NHS Foundation Trust,
Denmark Hill,
SE1 9RT,
London,
United Kingdom
Email address: kfan@nhs.net

Key words: Artificial Intelligence, Machine Learning, Automated Classification, Oral Cancer, Oral Potentially Malignant Disorder

Supplementary Information

Supplementary Table S1: Definitions of key terms relevant to this systematic review. TP, true positive; FP, false positive; TN, true negative; FN, false negative; Grad-CAM, Gradient-weighted Class Activation Mapping

Term	Definition
General	
Machine Learning	The ability of a machine to *learn* information and draw inferences from patterns within data without explicit programmed instruction.
Artificial Intelligence	The domain of computer science concerned with the development of computer systems able to perform tasks usually requiring human intelligence.
Deep Learning	A subfield of machine learning involving the use of complex neural networks with multiple layers (>3) to allow automatic feature selection from unstructured input data.
Neural Network	AI architectures comprising multiple algorithms in interconnected layers inspired by their biological counterparts, that allow complex feature selection and pattern recognition.
Transfer Learning	The application of a machine learning algorithm trained on one task to solve a separate but related task.
Augmentation	A technique used to artificially create new training data from existing training data through transformation.
Supervised Learning	Subcategory of machine learning where algorithms are trained on labelled datasets to classify unlabelled data. The labelled standard is the ground truth.
Unsupervised Learning	Subcategory of machine learning where algorithms detect patterns within data without pre-labelling. Patterns within unlabelled data are used for clustering or association.
Performance metrics	
Sensitivity (TP/(TP+FN))	The ability of a test to correctly identify patients with a disease. Also known as recall more generally.
Specificity (TN/(TN+FP))	The ability of a test to correctly identify people without a disease.
Precision (TP/(TP+FP)	The fraction of positive test cases that truly are positive.
F_1 Score (Sørensen–Dice coefficient)	The harmonic mean of precision and recall that provides a measure of accuracy of binary classification systems. A value of 1.0 indicates perfect precision and recall, whereas 0 indicates that precision or recall is 0. $$F_1 = \frac{TP}{TP + \frac{1}{2}(FP + FN)} = 2.\frac{precision \times recall}{precision + recall}$$
Accuracy	The ability of a classification system to differentiate correctly between healthy and diseased cases. $$\text{Accuracy} = \frac{TP + TN}{TP + TN + FP + FN}$$
Grad-CAM[9]	A method of visualising and optimising performance of an algorithm by producing a coarse localisation map highlighting regions of interest within an image source important for prediction.

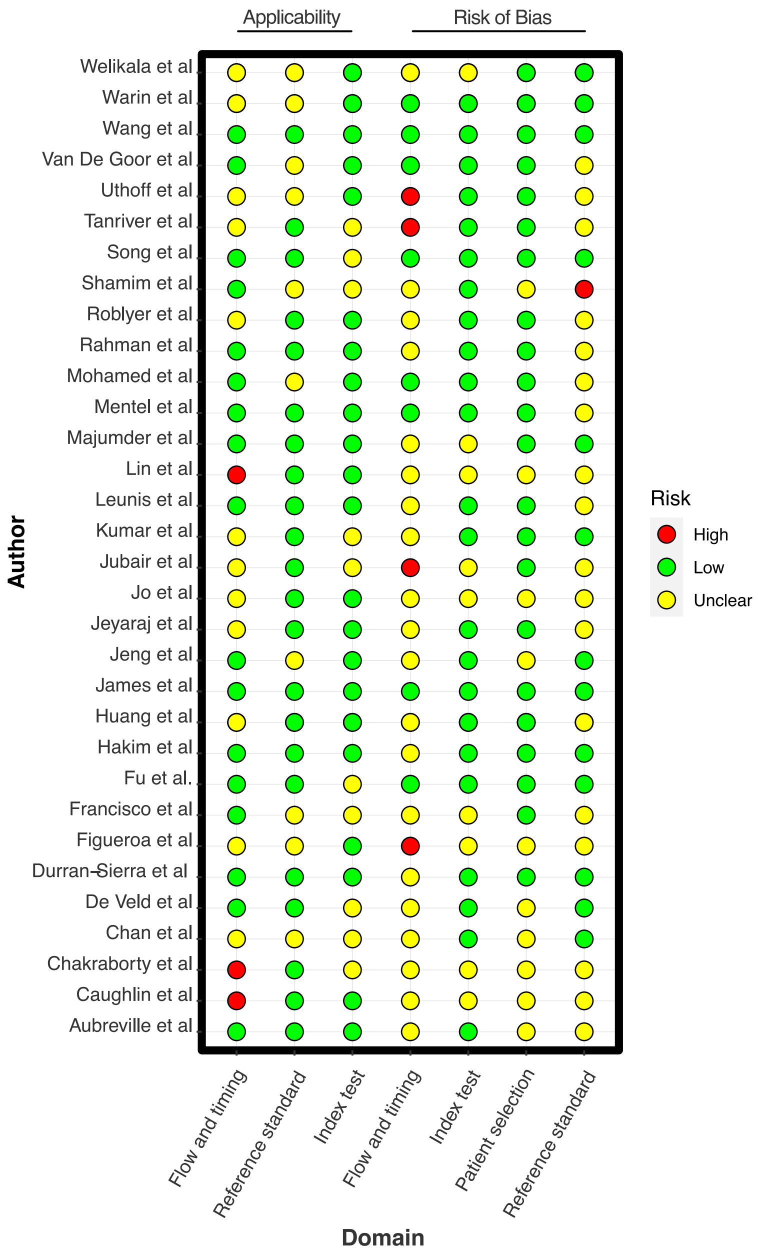

Supplementary Figure S1: Traffic-light plot of QUADAS-2 risk of bias assessment tool for individual studies

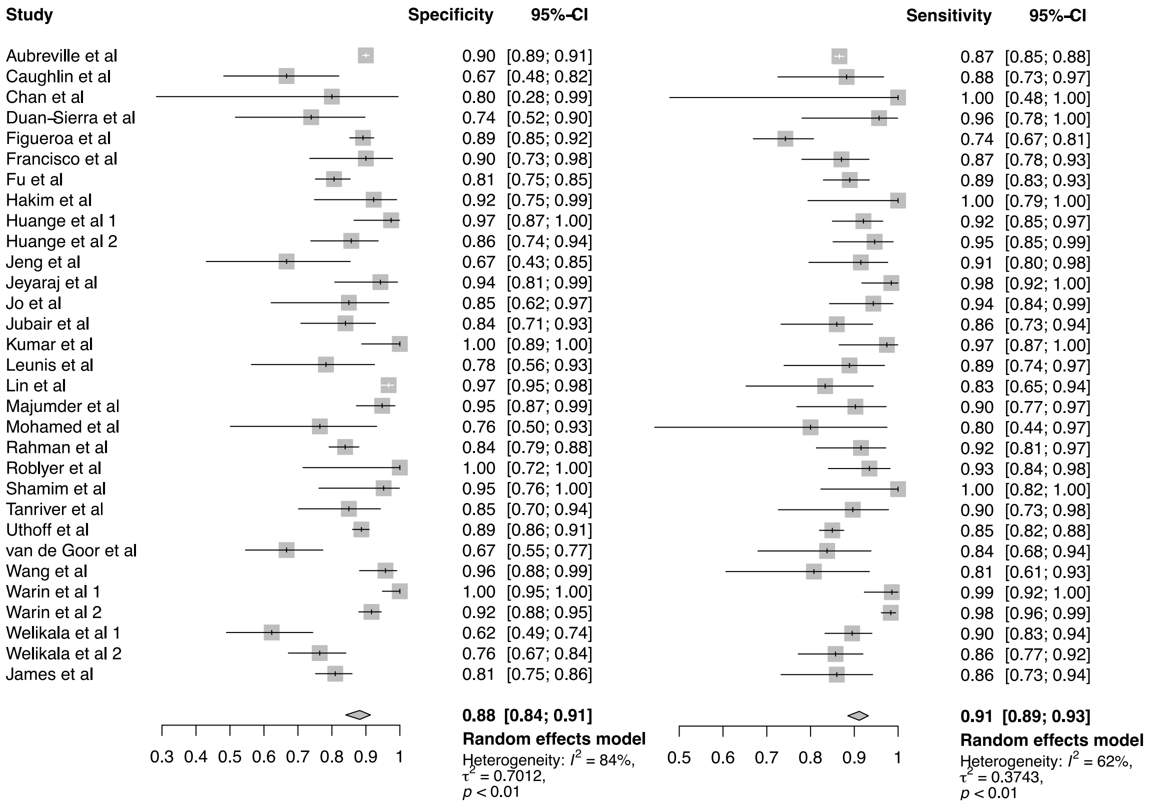

Supplementary Figure S2: Forest plots of both specificity and sensitivity of studies included in quantitative analysis. Values in brackets indicate 95% confidence intervals. These plots are derived through univariate random-effects meta-analysis of sensitivity and specificity and were thus used only to inform on study heterogeneity.

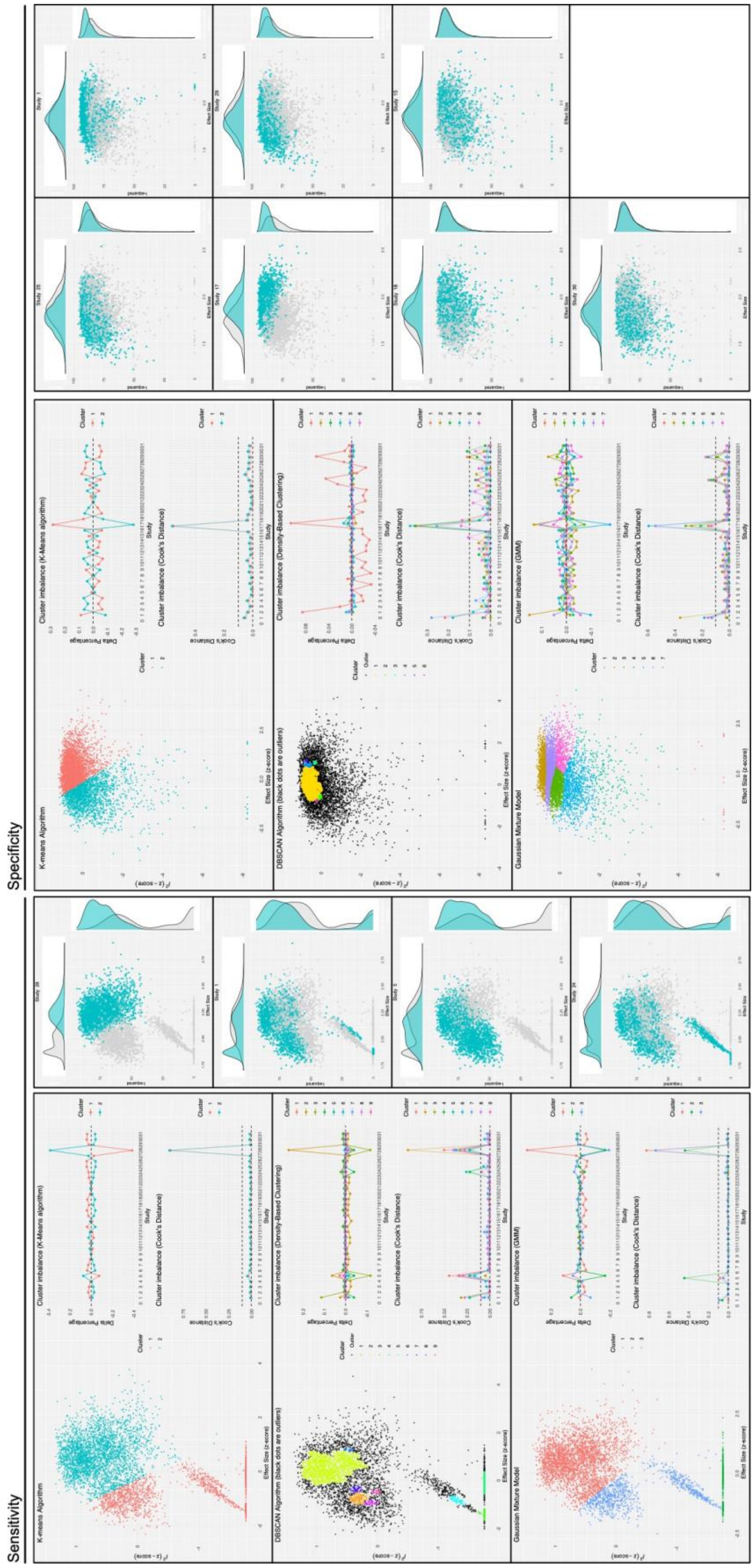
Sensitivity
Specificity
K-means Algorithm
Cluster imbalance (K-Means algorithm)
Cluster imbalance (Cook's Distance)
DBSCAN Algorithm (black dots are outliers)
Cluster imbalance (Density-Based Clustering)
Gaussian Mixture Model
Cluster imbalance (GMM)
Effect Size (z-score)
Delta Percentage
Cook's Distance
Study
Cluster

Supplementary Figure S3: GOSH plot of sensitivity data (left) and specificity data (right) with outcome of *k*-means clustering, Density-Based Spatial Clustering of Applications with Noise (DBSCAN), and Gaussian unsupervised algorithms. Potentially influential studies are determined as those with a leverage 3 times above the mean in any generated cluster. A complete explanation of GOSH diagnostics is provided by Harrer M and Ebert D, 2021.

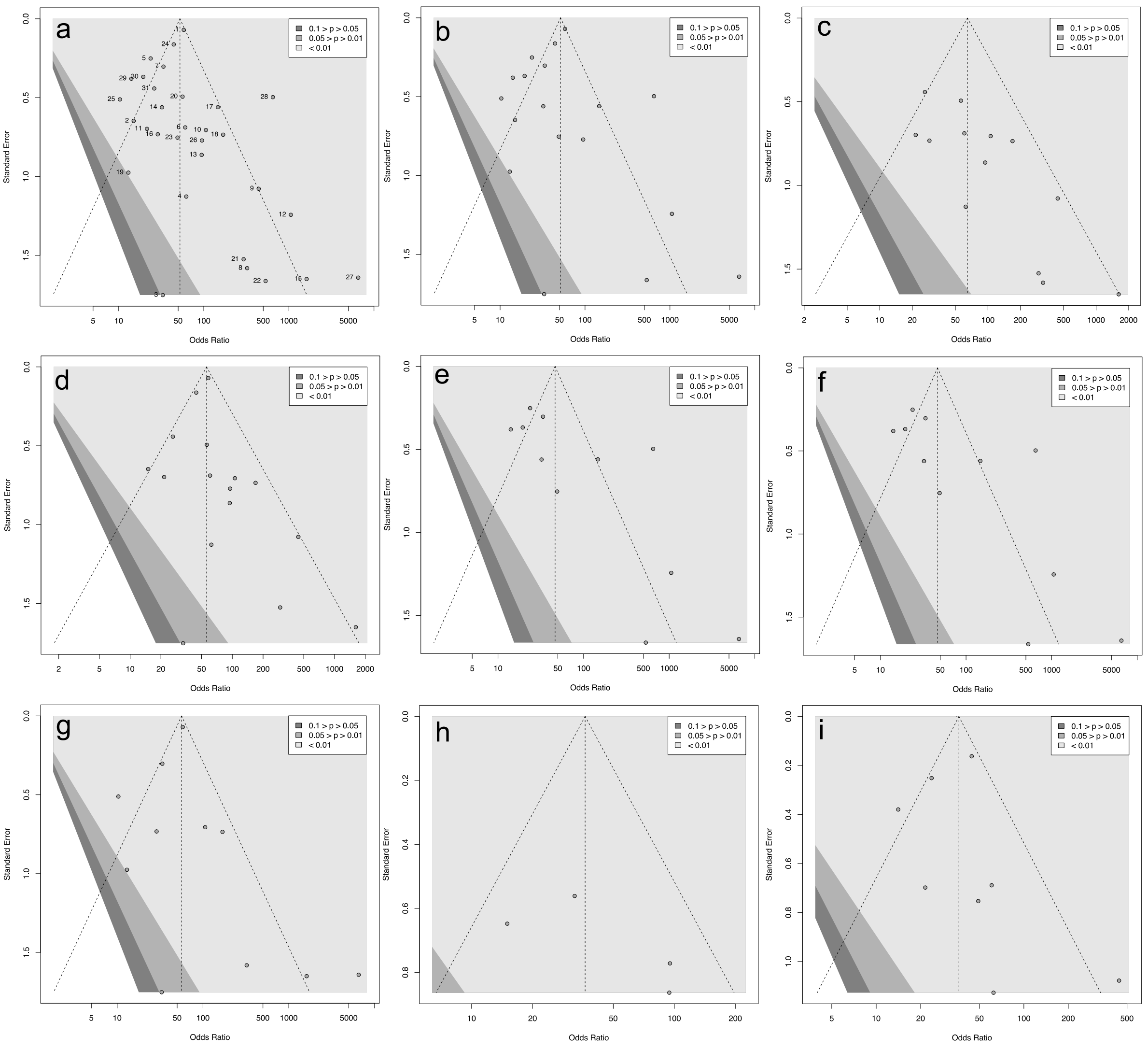

Supplementary figure S4: Funnel plots used to assess for small study effects; a, all studies; b, AI type = modern; c, AI type = classical; d, modality = optical imaging; e, modality = clinical photographs; f, modality = volatile compounds; g, classification = OSCC vs healthy; h, classification = OSCC/OPMD vs benign; I, classification = OSCC/OPMD vs healthy.

Supplementary table S2: Outcome of assessment for publication bias.

Group	Sample	Main analysis – Diagnostic OR [95% CI]	Linear regression test for Funnel plot asymmetry (Egger's test)	Trim and Fill method, OR [95% CI], n imputed studies	Test of small-study effects, Q-Q'(df), p-value	Test of residual heterogeneity beyond small study effects, Q'(df), p-value
Overall	NA	60.45 [39.87; 94.02]	t(29) = 0.55, p = 0.59	45.79 [27.67;75.78], n = 5	Q-Q'(1) = 1.2, p = 0.27	Q'(29) = 115.4, p <0.0001
AI type	Modern	53.19 [28.05; 100.85]	t(16) = -0.21, p = 0.838	53.19 [28.05; 100.95], n = 0	Q-Q'(1) = 0.26, p = 0.61	Q'(16) = 96.42, p <0.0001
	Classical	70.44 [41.09; 120.77]	t(11) = 3.55, p = 0.0045	45.70 [24.79; 84.24], n = 5	Q-Q'(1) = 10.28, p = 0.0013	Q'(11) = 8.96, p = 0.63
Modality	Clinical photographs	87.01 [31.51;240.30]	t(9) = 2.62, p = 0.0279	30.32 [7.74; 118.74], n = 4	Q-Q'(1) = 31.10, p <0.0001	Q'(9) = 40.81, p <0.0001
	Optical imaging	53.79 [43.19; 67.00]	t(14) = 0.67, p = 0.511	50.88 [39.76; 65.10], n = 4	Q-Q'(1) = 0.65, p = 0.42	Q'(14) = 38.34, p <0.0005
	Volatile compounds	18.95 [7.66; 46.88]	t(2) = 2.11, p = 0.169	15.10 [4.89; 46.66], n = 1	Q-Q'(1) = 3.46, p = 0.06	Q'(2) = 1.55, p = 0.46
Comparison	OSCC vs Healthy	68.00 [27.75;166.57]	t(9) = 0.25, p = 0.811	47.35 [16.07; 139.54], n = 2	Q-Q'(1) = 0.23, p = 0.0002	Q'(9) = 33.84, p <0.0001
	OSCC/OPMD vs Benign	40.41 [17.30; 94.39]	t(2) = 1.42, p = 0.292	40.41 [17.30; 94.39], n = 0	Q-Q'(1) = 2.34, p = 0.126	Q'(2) = 2.33, p = 0.31
	OSCC/OPMD vs Healthy	34.93 [21.00; 58.11]	t(6) = 0.34, p = 0.746	31.39 [17.84; 55.23], n = 1	Q-Q'(1) = 0.33, p = 0.57	Q'(6) = 17.01, p = 0.009
